# Tumor necrosis factor related apoptosis inducing ligand (TRAIL) regulates deubiquitinase USP5 in tumor cells

**DOI:** 10.18632/oncotarget.27196

**Published:** 2019-10-08

**Authors:** Harish Potu, Malathi Kandarpa, Luke F. Peterson, Nicholas J. Donato, Moshe Talpaz

**Affiliations:** ^1^ Department of Internal Medicine, University of Michigan Rogel Cancer Center, Ann Arbor, MI 48109, USA; ^2^ Center for Scientific Review, National Institutes of Health, Bethesda, MD 20892, USA

**Keywords:** USP5, ubiquitin, TRAIL, DUB inhibitor, G9

## Abstract

The tumor necrosis factor-related apoptosis-inducing ligand (TRAIL) pathway has emerged as a cancer therapeutic target. However, clinical trials have proven that most human cancers are resistant to TRAIL. We show that exposure to recombinant TRAIL resulted in the accumulation of ubiquitinated proteins and free ubiquitin polymers, suggesting a link between TRAIL and the ubiquitin (Ub)-proteasome pathway. TRAIL treatment in cancer cells reduced the activity and cleavage of USP5, a deubiquitinase (DUB) previously shown to target unanchored Ub polymers and regulate p53-mediated transcription. TRAIL was effective in suppressing USP5 activity and cleavage in TRAIL-sensitive cells but not resistant cells. Knockdown of USP5 in TRAIL-resistant cells demonstrated that USP5 controls apoptotic responsiveness to TRAIL. USP5 cleavage and ubiquitination were blocked by caspase-8 specific inhibitors. A small-molecule USP5/9× inhibitor (G9) combined with TRAIL enhanced apoptosis and blocked colony growth in highly TRAIL-resistant cell lines. Finally, USP5 protein levels and activity were found to be frequently deregulated in TRAIL-resistant cells. Together, we conclude that activated TRAIL enhances USP5 activity and induces apoptosis in TRAIL-sensitive and -resistant cells. We also suggest that USP5 inhibition may be effective in inducing apoptotic thresholds to enhance responsiveness to TRAIL.

## INTRODUCTION

Tumor necrosis factor (TNF)-related apoptosis-inducing ligand (TRAIL) appears to be a promising candidate for cancer therapeutics because of its selective cytotoxic effect on cancer cells while sparing normal cells [1–3]. TRAIL induces apoptosis by binding to two death receptors DR-4 (TRAIL-R1) and DR-5 (TRAIL-R2). This leads to the recruitment of the adaptor protein, Fas-associated death domain (FADD), which in turn recruits the initiator caspase, caspase-8, resulting in the formation of the death-inducing signaling complex (DISC). Within the DISC, FADD and caspase-8 recruitment leads to the activation of caspase-8 which can subsequently activate downstream effector caspase-3 and leads to apoptosis [4–6]. However, a number of cancer cells are resistant to TRAIL, especially solid tumors such as glioma, pancreatic and breast [7–9] Therefore, unveiling resistance mechanisms to TRAIL-induced apoptosis, might be beneficial in finding molecules that may play critical roles in resistance process and provide opportunities to overcome TRAIL resistance.

Ubiquitination of proteins can regulate both pro-survival and pro-apoptotic signals, including the TRAIL pathway [10, 11]. Protein ubiquitination is sequentially mediated by three enzymes: the ubiquitin-activating enzyme (E1), ubiquitin-conjugating enzyme (E2), and ubiquitin ligase (E3) that controls substrate specificity [6, 12]. The Ubiquitin proteasome system leads to the degradation of the ubiquitinated proteins. Proteasome inhibitors such as bortezomib could dramatically sensitize multiple myeloma cells [13], and a variety of human and mouse solid tumor cells to the apoptotic effects of TRAIL [13, 14]. Other ubiquitin ligases control the apoptosis pathway such as, A20 E3 ligase mediates RIP1 ubiquitination through a K63-linked polyubiquitin chain that binds caspase-8 and inhibits TRAIL-induced apoptosis in human glioblastoma [15]. The E3 ligase, ITCH, ubiquitinates caspase-8 inhibitor, FLIP, inducing its proteasomal destruction and enhancing proapoptotic TNFα signaling [16]. In Cylindromatosis (CYLD), a deubiquitinase (DUB), removes the polyUB chains from RIP1 and promotes caspase-8 cleavage mediating TNFα-induced apoptosis [17]. By contrast, caspase-8 ubiquitination at its C-terminus by a CUL3-based E3 ligase complex promotes caspase-8 activation and apoptosis [18].

We previously noted that knock-down of USP5 led to the activation of p53 and FAS expression in melanoma [19], indicating that DUBs likely play a role in the extrinsic pathway of apoptosis. Here, we sought to investigate the role of deubiquitinases (DUBs) in different TRAIL-sensitive and resistant cancer cell lines. We show the regulation of USP5 by TRAIL and how USP5 knock-down re-sensitizes TRAIL-resistant cells. We first noted a change in total ubiquitinylated protein content and unanchored Ub chains in melanoma and other cancer cell lines treated with TRAIL but not in TRAIL-resistant cells. We performed unbiased assessment of DUB activity in TRAIL-treated and control cells and determined that some DUBs were affected, including USP5. TRAIL consistently inhibited USP5 activity and cleaved USP5, which could be blocked by specific caspase-8 inhibitors. Initially, we used specific knockdown (KD) of this subset of DUBs to examine their role in melanoma and other resistant cells and determined that USP5 KD -sensitizes cells to TRAIL. Since USP5 KD induces apoptosis in TRAIL-resistant cell lines in the presence of TRAIL. Gene array analysis showed that KD of USP5 induces death receptors (DR4, DR5). Inhibition of USP5 activity by small molecules may be an attractive strategy for improving therapy in TRAIL resistant tumors. Towards this goal, we used a small molecule DUB inhibitor (G9) [20, 21] with activity against USP5/USP9× and demonstrate improved apoptotic activity in TRAIL-sensitive and resistant cells.

## RESULTS

### TRAIL treatment leads to increased ubiquitination in cancer cells

**Figure 1:**
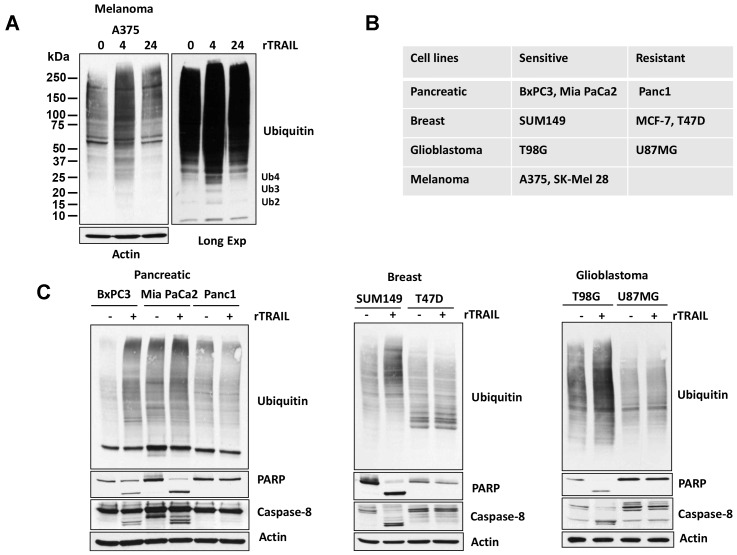
TRAIL regulates Ubiquitin pathway. (**A**) A375 melanoma cells were treated with 100 ng rTRAIL for 4 and 24 hr before whole cell lysates were resolved on SDS-PAGE high-percent cross-linked gels and subjected to immunoblotting for total ubiquitin. The mobility of di-, tri- and tetra-Ub is denoted (long exp). (**B**) Table of TRAIL-sensitive and -resistant cell lines. (**C**) TRAIL-sensitive and - resistant cells were treated with 100 ng rTRAIL for 4 hr before whole cell lysates were resolved on SDS-PAGE high-percent cross-linked gels and subjected to immunoblotting for total ubiquitin. Cell lysates were immunoblotted for the Caspase-8 and PARP protein indicated and actin served as a loading control.

To determine whether TRAIL affects post-translational signals other than apoptosis, we assessed kinetics of total protein ubiquitination in TRAIL-treated melanoma cells. TRAIL increased protein ubiquitination in a TRAIL-responsive melanoma cell line (Figure 1A). In addition to protein ubiquitination, Ub polymers (Ub_2–4_) increased, as determined by longer exposure of immunoblots. These changes were reminiscent of increased Ub polymers in cells with inhibition/knockdown of USP5, a DUB, with activity against unanchored poly-ubiquitin chains [22]. We further assessed if this ubiquitination profile is also evident in TRAIL-sensitive and -resistant pancreatic, breast and glioblastoma cell lines (Figure 1B). As hypothesized, the sensitive cell lines demonstrated increased total ubiquitination content which correlated with PARP and caspase 8 cleavages, but not resistant cell lines. (Figure 1C).

### TRAIL and FAS regulate USP5 activity

**Figure 2:**
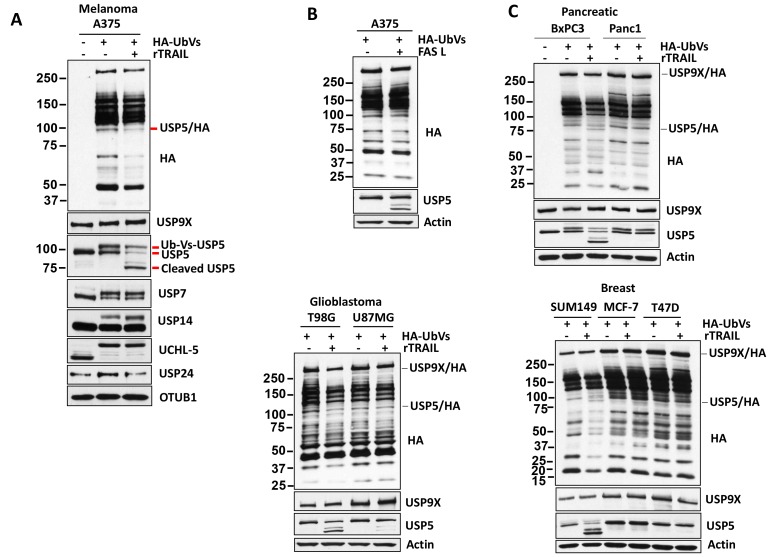
TRAIL regulates USP5 activity. (**A**) Melanoma cells were incubated with concentration (100 ng) of TRAIL for 4 hours before DUB activity was assessed in lysates by HA-UbVS labeling followed by HA blotting (top). The migration of Usp5 and HA-labeled Usp5 (appears as a doublet) is detected by USP5 immunoblotting (below) and immunoblotted for the USP9X, USP7, USP14, UCHL-5 protein indicated and OTUB1 served as a loading control. (**B**) A375 melanoma cells were incubated with concentration (50 ng) of FASL for 6 hours. DUB activity was assessed in lysates by HA-UbVS labeling followed by HA blotting (top). The identity of an HA-labeled DUB, USP5, is denoted. (**C**) TRAIL-sensitive (BxPC3, T98G, SUM149) and resistant (Panc1, U87MG, MCF-7, T47D) cells were incubated with concentration (100 ng) of TRAIL for 4 hours, and DUB activity was assessed in lysates by HA-UbVS labeling followed by HA blotting. HA-labeled USP9X and USP5 are highlighted. Actin served as a loading control.

The increase in polyubiquitin chains and ubiquitinated proteins was reminiscent of observations seen after USP5 inhibition or knockdown [22]. To determine whether TRAIL treatment affects DUB activity, lysates were labeled with HA-Ub-Vinylsulfone (HA-UbVS), an irreversible DUB inhibitor that covalently modifies active DUBs with HA-Ub. in addition, HA-UbVS labeling demonstrated a mobility-shift of the DUBs (Figure 2A). HA blotting demonstrated that TRAIL exposure altered USP5 activity and induced its cleavage in TRAIL-sensitive melanoma A375 cells. The identity of USP5 was confirmed based on its molecular mass, LC/MS of excised bands (data not shown) and direct immunoblotting with USP5 antibody (Figure 2A). TRAIL treatment did not alter the activity of DUBs: USP9×, USP7, USP14, USP24 and UCHL-5. OTUB1, another DUB, which was also not altered and served as a control for equal loading of proteins from the lysates. Similar results were obtained in pancreatic, glioblastoma and breast cancer cell lines as indicated. TRAIL completely reduced USP5 activity in TRAIL-sensitive cells (BxPC3, T98G, SUM149) but not in resistant cells (Panc1, U87MG, MCF-7, T47D) (Figure 2C). We also noted elevated basal activity and protein levels of USP9× and USP5 in TRAIL-resistant cell lines (U87MG, MCF-7 and T47D) (Figure 2C) (Supplementary Figure 1) compared to sensitive cells. We also examined if other TNF family proteins have the same effect as TRAIL and treated A375 (TRAIL sensitive) cells with FAS. Similar to TRAIL, FAS treatment induced the cleavage of USP5 while it but did not alter the DUB activity of USP9× (Figure 2B). Together, these results suggest that TRAIL and FAS regulate USP5 activity in sensitive cells but not in resistant cell lines.

### USP5 cleavage depends on caspase-8

**Figure 3:**
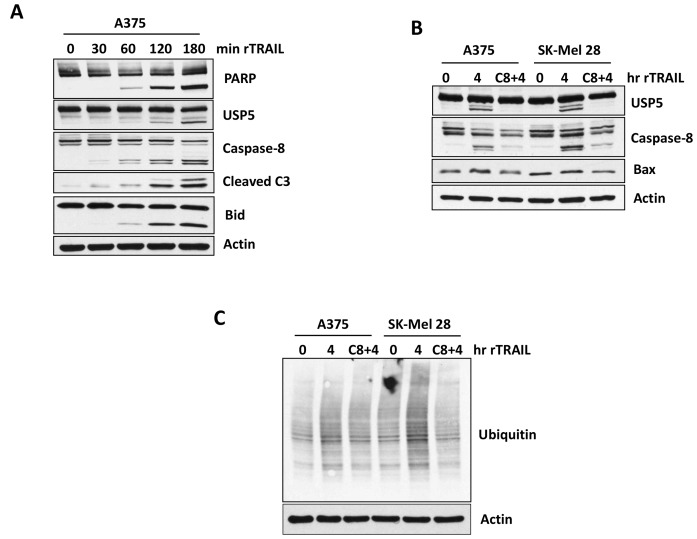
Caspases controls USP5 cleavage. (**A**) A375 melanoma cells were treated with 100 ng TRAIL for indicated time in minutes. Cell lysates were subjected to immunoblotting for the protein indicated, including USP5 cleavage. (**B**) A375 and SK-MEL28 melanoma cells (TRAIL sensitive) were treated first with Caspase-8 inhibitor (50 μM) for hour and then treated with 100ng of TRAIL for 4 hr. Cell lysates were immunoblotted for the protein indicated, including USP5 cleavage. (**C**) A375 and SK-MEL28 melanoma cells (TRAIL sensitive) were treated first with Caspase- 8 inhibitor (50 uM) for hour and then treated with 100 ng of TRAIL for 4 hr. Cell lysates were blotted for total ubiquitin protein. Actin served as a loading control.

Previously, proteome changes in response to TRAIL-mediated apoptosis was investigated in Jurkat T-cells with and without caspase inhibitors (z-VAD-FMK/pan-caspase) [23]. More than 650 cleaved proteins were identified by mass spectrometry (MS), including USP5 (DDLDAEA, SAADSIS) in TRAIL-treated but not in caspase-dependent cells (Supplementary Figure 2) [23]. To further assess the role of USP5 cleavage in the apoptotic response to TRAIL, A375 cells were treated with TRAIL for short intervals, and the onset of caspase 8, Bid and PARP cleavage were assessed. As shown in Figure 3A, TRAIL treatment led to caspase 8 cleavage, followed by BID and PARP cleavage (1–3 hours). We also noted cleavage of USP5, possibly as a consequence of caspase activation, but the significance of this modification is not yet known. Therefore, we further examined whether inhibition of caspase-8 could block USP5 cleavage in A375 melanoma cells. Pre-treatment with Z-IETD-FMK (caspase 8 inhibitor) for 1 hr significantly blocked USP5 cleavage after treatment with TRAIL for 4 hr (Figure 3B). Caspase 8 inhibition decreased caspase 8 cleavage/activation induced by TRAIL. Inhibition of caspase-8 also significantly reduced total ubiquitination after treatment with TRAIL in A375 melanoma cells (Figure 3C). In caspase-8-inhibited cells, these activities (ubiquitination and cleavage) were blocked, suggesting a prominent role for both USP5 and caspase-8 in the activation of TRAIL-mediated cell death. These data suggest that cleavage of USP5 by TRAIL treatment depends on caspase-8.

### USP5 regulates apoptotic response to TRAIL

**Figure 4:**
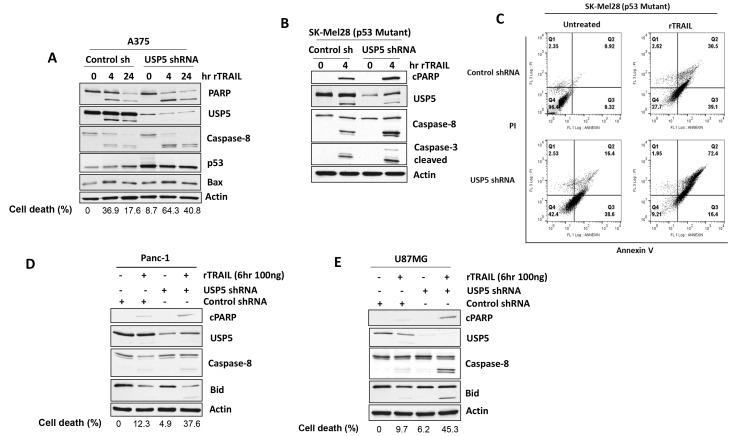
USP5 regulates apoptotic responsiveness to TRAIL. (**A**) A375 melanoma cells (TRAIL sensitive) were infected with control and USP5 shRNA lentiviral vectors, selected in puromycin and treated with TRAIL 100 ng for indicated hr. Cell lysates were immunoblotted for PARP, USP5, p53, BAX protein indicated and annexin V assessment for cell death (%). (**B**) SK-Mel28 (p53 mutant) melanoma cells were infected with control and USP5 shRNA lentiviral vectors, selected in puromycin and treated with TRAIL 100 ng for 4 hr. (**C**) Cell lysates were immunoblotted for the protein indicated and annexin V assessment for cell death (right). (**D**) Pancreatic (Panc1) cells (TRAIL resistant) were infected with control and USP5 shRNA lentiviral vectors, selected in puromycin and treated with TRAIL 100 ng for 6 hr. Cell lysates were subjected to immunoblotting for the protein indicated and annexin V assessment for cell death (%). (**E**) U87MG glioblastoma cells (TRAIL resistant) were infected with control and USP5 shRNA lentiviral vectors, selected in puromycin and treated with TRAIL 100 ng for 6 hr. Cell lysates were immunoblotted for the protein indicated and annexin V assessment for cell death (%). Actin served as a loading control.

We first examined effects of USP5 knock down (KD) in A375 (sensitive) melanoma cells treated with TRAIL. USP5 KD was achieved using shRNA targeted to USP5. USP5 KD increased apoptotic response (Bid and PARP cleavage) to TRAIL in melanoma cells (Figure 4A). USP5 KD also resulted in increased levels of p53 protein, perhaps by protecting p53 from proteasomal degradation as previously described [19, 22]. These apoptotic signaling events also occur in other TRAIL-responsive melanoma cells expressing mutant p53 (SK-Mel28) (Figure 4B) and apoptosis (Figure 4C) (Supplementary Figure 3). To determine whether USP5 also regulates apoptotic response in TRAIL-resistant cell lines, USP5 KD and control cells were treated with TRAIL. TRAIL induced apoptosis in all cells, but had limited impact on PARP cleavage in a pancreatic cell line (Panc1) (Figure 4D). In USP5 KD cells, TRAIL treatment further enhanced cleavage of caspase-8 which correlated with PARP and BID cleavage. Similar results were obtained in a glioblastoma line (U87MG) with USP5 KD and TRAIL treatment (Figure 4E), suggesting that USP5 can regulate the apoptotic response to TRAIL in resistant cell lines.

### DUB inhibition overcomes acquired resistance to TRAIL

**Figure 5:**
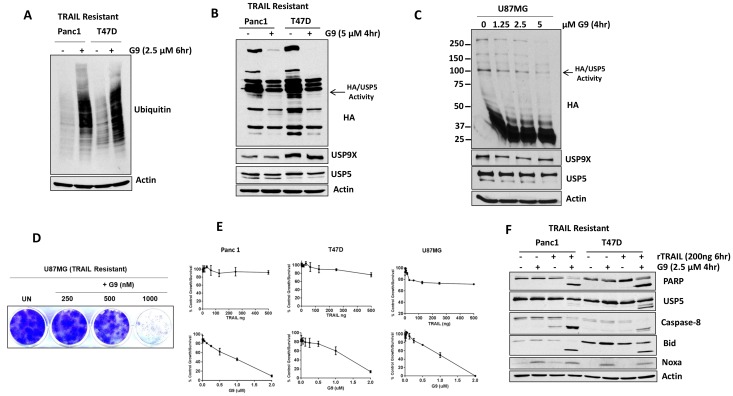
USP5 inhibition overcomes acquired resistance to TRAIL. (**A**) TRAIL-resistant cells lines were treated with G9 (+) for 6 hr, and whole cell lysates were resolved on SDS-PAGE high-percent cross-linked gels and subjected to immunoblotting for total ubiquitin. Actin was used as a loading control. (**B**) TRAIL-resistance cells lines were incubated with the indicated concentration of G9 for 4 hours before DUB activity was assessed in lysates by HA-UbVs labeling followed by HA blotting (top). USP5 immunoblotting resolves the migration of USP5 and HA-labeled USP5 (appears as a doublet). (**C**) TRAIL-resistance glioma (U87MG) cells lines were incubated with the indicated concentrations of G9 for 4 hours before DUB activity was assessed in lysates by HA-UbVs labeling followed by HA blotting (top). The identity of HA-labeled DUB USP5 is denoted. (**D**) Colony growth was assessed by crystal violet staining of glioma U87MG cells treated with G9 at the indicated concentrations for 21 days in standard 2D culture. (**E**) TRAIL-resistant cell lines were treated with TRAIL (upper) and G9 (lower) at the indicated concentrations for 72 hr, and cell growth was assessed by MTT assay. The results represent the average ± S.D. of triplicate assays. (**F**) Pancreatic (Panc1) and breast (T47D) cells (TRAIL resistant) were treated with TRAIL 200 ng for 6 hr alone, G9 for 4 hr alone, or the combination, and cell lysates were subjected to immunoblotting for the protein indicated.

To determine whether the combination of DUB inhibition and TRAIL can regulate the cellular response to TRAIL in resistant cell lines, we assessed total protein ubiquitination in TRAIL-resistant pancreatic (Panc1) and breast cancer (T47D) cells in the presence of a DUB (G9) inhibitor. Cells were treated with a small molecule USP5/9× inhibitor G9 and cell lysates were immunoblotted for total ubiquitin and DUB activity. G9 treatment increased protein ubiquitination (Figure 5A) and reduced USP5 and USP9× activity in both cell types (Figure 5B, middle). Similarly, in a glioblastoma cell line, G9 decreased USP5 activity in a dose-dependent manner (Figure 5C, right). G9 was also highly effective in inhibiting colony growth in TRAIL-resistant glioma U87MG cells (Figure 5D) and led to greater inhibition of cell growth compared with TRAIL treatment alone (Figure 5E). We also assessed the apoptotic activity of G9 in combination with TRAIL. Cells were treated with G9 alone, TRAIL alone, or in combination (G9 was added for the final 4 hours of TRAIL treatment). G9 or TRAIL alone had minimal impact on caspase activation and USP5 cleavage, while G9 increased PARP cleavage (Figure 5F). The combination treatment resulted in activation of extrinsic caspase cascades, and induced cleavage of Bid and PARP in TRAIL-resistant cell lines (Figure 5F). These results suggest that USP5 cleavage in TRAIL-resistant cell lines can modulate apoptosis levels in response to DUB inhibition.

## DISCUSSION

TRAIL is selectively cytotoxic to cancer cells and therefore is an attractive candidate for specific targeting of cancer cells while sparing normal cells. However, analysis of a large panel of established human cancer cell lines showed that 40-50% of cancer cell lines were either partially or completely resistant to TRAIL [24]. TRAIL resistance is a major limitation for its clinical use in the treatment of human cancers [25–29]. The cause of TRAIL resistance is under intense investigation, and many possible mechanisms have been proposed including protein degradation by proteasome pathway. We assessed the role of ubiquitin proteasome cascade by TRAIL exposure. TRAIL consistently altered protein ubiquitination in TRAIL-sensitive cancer cells but not in the TRAIL-resistant cells (Figure 1C). TRAIL also led to the accumulation of ubiquitin polymers, which is reminiscent of previous observations in cells with DUB knockdown or DUB-inhibitor treatment [20, 21]. Further assessment confirmed that TRAIL inhibited DUB activity. Labeling of DUBs using HA-Ub-Vs showed that at least 4-5 DUBs were consistently suppressed or inhibited by TRAIL in sensitive cells. This labeling can only recognize USP (ubiquitin specific protease) family members, and thus TRAIL may also affect other ubiquitin and DUB family proteins including E1, E2 and E3 ligases; in fact, cleavage of the E3 ligase RNF31 was shown to be TRAIL dependent [30]. The few DUBs inhibited by TRAIL may not be direct targets and should be further investigated. We identified that TRAIL consistently suppressed the enzymatic activity of USP5 and led to its cleavage in sensitive cell lines but not in TRAIL-resistant cells. Levels of intrinsic USP5 activation and expression differed in TRAIL-sensitive and -resistant cells and were diminished by DUB inhibitor G9 treatment.

USP5 contains a zinc finger, two ubiquitin-associated domains and a Ubiquitin specific protease (USP) domain. Of note, the 767, 782 cleavage sites that were discovered previously are located at the C-terminus of the USP domain [26]. We speculate that the cleavage of USP5 results in the separation of its USP domain, and therefore cleaved fragments are able to fully induce global ubiquitination. These studies uncovered links between TRAIL and USP5 cleavage in cancer cells, but whether USP5 regulates these cleavages directly or indirectly requires further investigation. Based on gene expression array analysis USP5 KD promotes expression of death receptors DR4 and DR5 (data not shown), which may at least partly underlie the high sensitivity of cells to USP5 inhibition. Since a broad range of tumor types express DR4 and DR5 [31], those regulated by DR4, DR5 may be good targets for USP5 inhibition. Indeed, previous reports have shown that stabilization of DR4 and DR5 induces apoptosis or high sensitivity to TRAIL [32–34]. Additional studies are needed to confirm this. These results uncover a new link between ubiquitin, death-receptor expression and apoptotic signaling, implicating potential biomarkers for TRAIL-based cancer therapy.

The therapeutic potential for targeting USP5 in combination with TRAIL seems high. However, this approach has not yet been tested, primarily due to the limited number of USP5 inhibitors available. To address that deficiency, we modified a small molecule partially selective DUB inhibitory (WP1130) [35] to make it more amenable pharmacological agent. The resulting compound G9 inhibited both USP9× and USP5 activities, and induced p53 and FAS levels (Figure 5B) [19]. G9 also induced apoptosis, which was amplified in TRAIL-resistant cells (Figure 5F). Overall, our results suggest that TRAIL controls the activity of specific DUBs including USP5 and USP9 ×. USP5 controls poly-ubiquitin levels and induces p53 and FAS, lowering apoptotic thresholds in melanoma [19]. In conclusion, the data presented here suggest that USP5 inhibits TRAIL-induced apoptosis. Thus, therapies that target USP5 activity may diminish TRAIL resistance and enhance the therapeutic efficacy of TRAIL-targeted therapies in human cancers. Dual TRAIL/DUB inhibition or mono-therapy with DUB inhibitors may be effective strategies for treating TRAIL-resistant tumors.

## MATERIALS AND METHODS

### Cell lines

A375, SK-Mel28 (melanoma), U87MG, T98G (glioma) cells were primarily maintained in Dulbecco’s Modified Eagle’s Medium (DMEM). Panc1, Mia PaCa2 (pancreatic) cells were cultured in DMEM, Glutamax. Breast cell lines HME, MCF10A were primarily maintained in DMEM: F12 (5% horse serum, insulin 5μg/ml, hydrocortisone 1 μg/ml, EGF 1 μg/ml, cholera toxin 1 μg/ml), MCF7 and T47D cells were cultured in medium MEM (insulin). HS578T and MDA-MB231 cells were primarily maintained in DMEM. SUM149 cells were cultured in medium F12 (insulin, hydrocortisone). All media was supplemented with 10% heat-inactivated FBS (Atlanta Biological), 2 mM L-glutamine and 1% penicillin/streptomycin (GIBCO).

### Chemical reagents

G9 was synthesized and provided by Evotec (UK), (Abingdon Oxfordshire, UK). Other reagents used in this study were obtained from the following sources: hemagglutinin-tagged ubiquitin vinyl methyl sulfone (HA-UbVS; Boston Biochem); recombinant TRAIL (a kind gift of Dr. Shaomeng Wang, University of Michigan, USA). All reagents were reconstituted and stored frozen as 10 mM stock solutions.

### shRNA-mediated gene silencing

Melanoma, pancreatic and glioblastoma cells were infected with the lentiviral expression system for short hairpin RNA (shRNA) for USP5 silencing, pGIPZ Control and pGIPZ-USP5 [19] were obtained from Open Biosystems. HEK293T cells were transfected with the lentiviral packaging vectors pMD2. G and psPax2 (Addgene) together with the shRNA vectors to produce virus using Poly Fect as described by the manufacturer (QIAGEN). The medium was changed to DMEM with 10% fetal bovine serum and after 48 hours, and the viral supernatant was collected with 2 mL of viral supernatant containing 4 μg/mL of Polybrene (Sigma-Aldrich). Two days after infection, the medium was changed and 1 μg/mL of puromycin was added. After puromycin selection (5 days), viable cells were recovered; USP5 levels were examined by immunoblotting. Those with stable reduction of USP5 were used to assess to analyze apoptotic sensitivity to TRAIL and other agents.

### Crystal violet colony staining

Equal numbers of viable U87MG (glioblastoma) cells were grown in 6 well plates for 3 weeks and subjected to crystal violet stain with 3.7% paraformaldehyde (PFA), 0.05% Crystal Violet in distilled water for 20 min at room temperature.

### DUB-labeling assays

Cells were lysed in DUB buffer (50 mM Tris pH 7.2, 5 mM MgCl_2_, 250 mM sucrose, proteasome inhibitors, 1 mM NaF and 1 mM PMSF) for 10 minutes at 4° C, followed by brief sonication. The lysates were centrifuged at 20,000 *g* for 10 minutes, and the supernatant was used for DUB labeling. Equal amounts of lysate (20 μg) were incubated with 2 μM of HA-UbVS [36] for 75 mins at 37° C, followed by boiling in reducing sample buffer and resolving by SDS-PAGE. HA immunoblotting was used to detect DUB labeling.

### Lysate preparation and western blotting

Total cell lysates were prepared by sonicating and boiling cell pellets in 1× Laemmli reducing sample buffer. To prepare detergent-soluble lysates, cells were lysed in cold isotonic lysis buffer (10 mM Tris-HCl, pH 7.5, 0.1% Triton X-100), 150 mM NaCl, with proteases inhibitor cocktail and 1 mM PMSF for 15 minutes on ice and centrifuged for 10 minutes at 20,000 g. The clarified supernatant was used as the detergent soluble cell fraction. Equal volumes of cellular lysate or equal protein amounts were electrophoresed on SDS-PAGE gels and transferred to nitrocellulose membranes. Proteins were detected by immunoblotting.

Antibodies used in this study were purchased from the following sources: anti-actin (Sigma-Aldrich); anti-ubiquitin clone P4D1, goat, anti-rabbit/mouse/rat IgG-conjugated horseradish peroxidase, USP7, USP5, USP9×, USP24, UCHL-5, USP14 and OUTB1 (Bethyl Laboratories; anti–poly (ADP-ribose) polymerase (PARP), Cleaved PARP (Asp214), Caspase8, Caspase3, BID, BAX (Cell Signaling Technology); anti-HA (clone 3F10; Roche Applied Science), anti-NOXA Santa cruz and CD95/Fas (Clone EPR5700; Epitomics)).

### MTT assay

Cells were seeded in a 96-well plate at 5,000 per well in the presence of the indicated concentration of compound for 3 days in a CO_2_ incubator at 37° C. MTT solution was added to each well for 2 hours at 37° C. The cells were then lysed in 10% SDS buffer, and absorbance at 570 nm was determined with a microplate reader.

### Statistical analysis

Data points are shown as the mean ± SD. Student’s *t* test was used to assess statistical performance using GraphPad Prism 6 and GraphPad InStat3.

### Apoptosis measurement

An Annexin V-fluorescein isothiocyanate (FITC) staining assay was performed as previously described [21]. The cells were seeded in six-well plates and exposed to rTRAIL as indicated for 4 and 24 hr. The cells were then trypsinized, washed with cold PBS, and stained with Annexin V-FITC for 10 min on ice. Positive cells were detected by flow cytometry.

## SUPPLEMENTARY MATERIALS


