# Supplementary material for: Tumor necrosis factor related apoptosis inducing ligand (TRAIL) regulates deubiquitinase USP5 in tumor cells
Source: Oncotarget. 2019 Oct 8;10(56):5745–54. doi: 10.18632/oncotarget.27196 (PMC6791380; doi:10.18632/oncotarget.27196)
Supplement: Supplementary file 1 [file oncotarget-10-5745-s001.pdf]

Tumor necrosis factor related apoptosis inducing ligand (TRAIL) regulates deubiquitinase USP5 in tumor cells

SUPPLEMENTARY MATERIALS

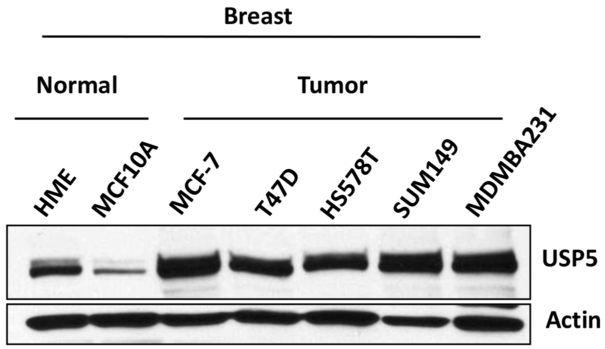

Supplementary Figure 1: Elevated expression of USP5 in tumor cells. Breast normal and tumor cell lysates were immunoblotted for the protein indicated. Actin served as a loading control.

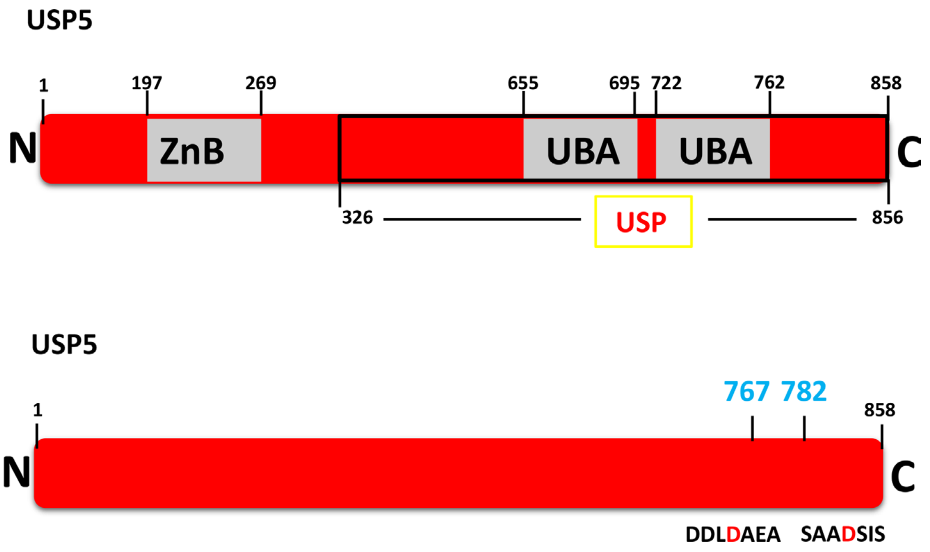

Supplementary Figure 2: TRAIL cleaves USP5 at C-terminal. Schematic diagram of the human USP5 protein with domains (top) showing the cleaved regions (bottom).

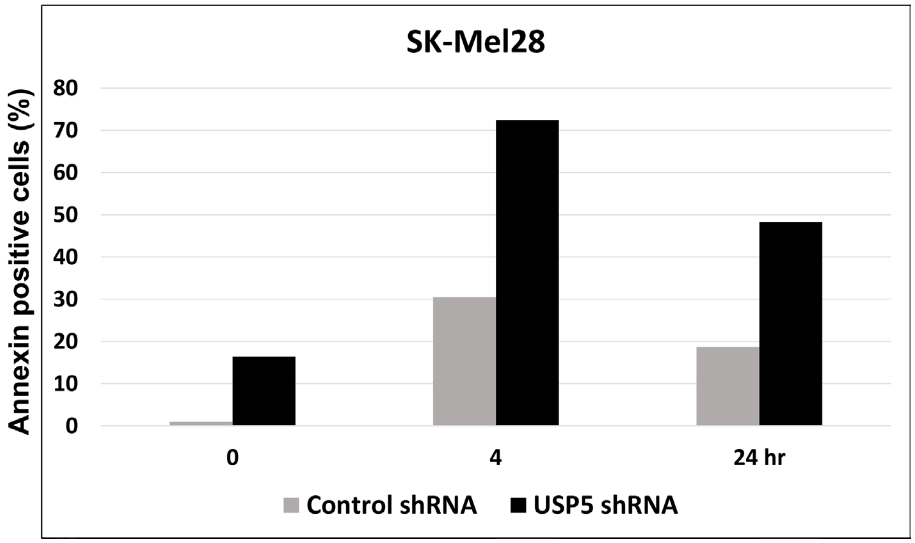

Supplementary Figure 3: USP5 KD induces cell death. Melanoma cells (TRAIL sensitive) were infected with control and USP5 shRNA lentiviral vectors, selected in puromycin and treated with rTRAIL 100 ng for indicated hr and cell death was assessed by annexin V-fluorescein isothiocyanate (FITC) staining assay.
